# So What? A Framework for Assessing the Potential Impact of Intervention Research

**DOI:** 10.5888/pcd10.120160

**Published:** 2013-01-17

**Authors:** Jonathan E. Fielding, Steven M. Teutsch

**Affiliations:** Author Affiliations: Jonathan E. Fielding, University of California, Los Angeles, Schools of Medicine and Public Health, and Los Angeles County Department of Public Health, Los Angeles, California.

Journals are full of studies of interventions with results that are statistically “significant” but lack guidance on the real importance of the work (1). We suggest that articles concerning clinical or population-health interventions should be accompanied by structured information about the potential health impact. This information should incorporate well-established metrics to expedite the translation of highly effective interventions into practice and reduce undue attention to studies of lesser consequence.

Although there are compelling reasons to address rare and unusual health conditions, research resources and health care costs in many countries are at an economic breaking point, and there should be more recognition of investments that yield the greatest health improvement. The importance of studies could be more readily apparent by characterizing a few key dimensions, potentially shaping both the content of journals and the choice and design of studies. Those dimensions reflect a framework that examines the benefits and harms of an intervention as it is likely to be used in real-world practice, a framework that is as applicable to clinical management as it is to public health programs and policies. Here we outline these dimensions.

## Quantitative Factors

**Burden of disease:** The burden of disease and injury should capture the overall health and economic consequences of the condition to the population as a whole as well as the disparities among subpopulations. Standard measures of burden include incidence, prevalence, mortality, years of potential life lost, attributable risk (ie, the burden of disease that is due to a risk factor), and measures of disparity among population groups. For overall health burden we recommend using quality-adjusted life years (QALYs) lost, which combines illnesses and deaths into a single metric. Although QALYs can be challenging to calculate and to understand, they are the best metric for making comparisons of different conditions. The largest components of economic burden are usually the costs of care, including medical care, out-of-pocket and caregiver costs, and productivity loss. These should be expressed on an absolute basis, that is, the total burden in the entire population (eg, total deaths in the total population, mortality rate), not just the relative burden within a specific disease category (eg, proportion of cancer deaths) or only a portion of the economic burden (eg, health care system costs, employer costs).

**Preventable burden:** Preventable burden is a measure of the adverse health outcomes that could be eliminated by implementing the interventions being studied compared with what is currently being done (ie, the avoidable incremental burden or reduction in disparities). Most simply, it is the product of the effectiveness of an intervention and the burden of disease. Effectiveness is the net health effect of an intervention when used in typical practice or population settings. At a population level, effectiveness should consider the extent to which the intervention will be used by the target population and unintended groups. It should be expressed in absolute terms so the anticipated population-level effect is apparent. By using QALYs, changes in both the quality of life and length of life are captured.

**Economic value**: The economic value should include the cost and cost-effectiveness of the intervention. Cost-effectiveness should be based on a comparison with existing interventions (incremental cost effectiveness), using a standard metric such as cost per QALY from the societal perspective (2) that would consider the value of the intervention in relation to other alternative expenditures. Any data from the intervention study comparing monetary benefits with monetary costs of the intervention should be included.

**Additional information from the study:** Evidence-based recommendations are made largely on the basis of the magnitude of effect of interventions and the certainty of the evidence. We are most interested in studies where effects were previously unknown (eg, in a new population) or where the evidence base has been insufficient to justify a recommendation. Studies to demonstrate that an intervention can work in a controlled setting (efficacy), such as randomized trials, and studies that demonstrate their effect in actual practice (effectiveness and generalizability) can provide important information. Investigators should indicate the extent to which their studies have enough power to enable new or different evidence-based recommendations overall or for a specific population. Other factors will influence the ultimate use of an intervention, but this suggested approach would at least outline the maximum benefit that could be derived from full implementation.

**Quantifying the impact:** This population perspective is inherently utilitarian, seeking to focus attention on interventions that deliver the greatest good for the entire population, particularly in general medical and public health publications. Implicit is the notion that resources are finite, and a key factor in rational resource allocation is the degree to which they maximize health overall. Much of the recent progress in clinical medicine has been the use of, for example, biomarkers that identify specific conditions with very costly specific therapies in small populations. We do not argue against these investments in research. Our concern is the current imbalance: important population interventions, including those that could target large disparities among subpopulations, are understudied and underused in relation to their potential population-health impact compared with more focused research of potential benefit to small numbers of people (3). As an example, sodium reduction in the food supply, which could have a modest effect but in a very large population, is understudied despite the potential for much greater health impact than many highly effective interventions for small populations (4).

## Challenge for Researchers

Presenting information in these terms may present a substantial challenge for many researchers. The challenge is not only the technical challenges of determining these metrics but also the challenge requiring them to find new ways to think about the value of their work — work whose value is often assessed by a peer group with keen interest in the same topics rather than its contribution to the public’s health. However, the demand for information about the potential health effect will encourage the development of methodologies and data sources and will provide impetus for training investigators in the value of information (5). The difficulty for researchers is compounded by the lack of standardized information on burden of disease in general and QALYs in particular; costs; and cost-effectiveness. These challenges could be addressed by increasing investment in registries and information systems to make this information more easily accessible. Incorporating these concepts into training of investigators would improve their ability to understand and communicate the value of their work. To do this work, researchers may need to partner with economists and epidemiologists with the technical skills to analyze and synthesize the literature and study data. Such partnerships can give researchers more insight into which aspects of their work have the greatest potential for health impact.

## Benefits of This Framework

Much as the structured abstract has provided clarity for readers about the content of journal articles, this framework can provide information that should increase attention to those studies of greatest value in improving the public’s health over the life course of the population. These data can benefit many different groups.

**Investigators:** An assessment of the potential effect of studies can guide selection of study topics and design likely to produce the most useful information.

**Funders:** Much as they should do for all types of funding, funders, including legislators and other government officials, using public resources work to ensure that research dollars are expended efficiently and effectively. Assuring that projects have substantial population-health benefit and that that benefit is then translated into improved health has been central to the National Institutes of Health Roadmap and Clinical Translation initiatives (6). This structured approach formalizes part of the selection process.

**Authors:** The standardized information will ensure that the author conveys the study’s importance to readers along with the description of the scientific work.

**Journal editors and reviewers:** Requiring this information with all journal submissions that assess an intervention could provide editors and reviewers with quantification of the importance of the work and could be valuable input into the manuscript review process. Reviewers should critique the authors’ estimates and assess the potential health impact as part of their standard review criteria.

**Journal readers**: Readers would benefit from having a better guide to why the article is important and what it contributes so they can identify the articles of greatest practical importance.

## Conclusion

Some initial steps need to be taken to develop this framework. A more detailed set of methods needs to be developed and pilot tested in existing publications. Building on that experience, journal editors, perhaps under the auspices of the International Committee of Medical Journal Editors, should convene an expert panel to formalize the types and forms of information that each manuscript of an intervention should contain. This would be analogous to the statements on reporting of clinical trials (CONSORT), observational trials (STROBE), or meta-analyses (MOOSE) (7–9).

It is critical to improve the value of clinical and population-health research and to enable rapid incorporation of the highest value interventions into practice. Providing standardized information on the impact of interventions can focus attention on those studies with the potential for the greatest population-health benefit. A consortium of journal editors, researchers, and funders should work to identify and develop the necessary resources and processes required.
